# Development of an Immunoassay for the Detection of Amyloid Beta 1-42 and Its Application in Urine Samples

**DOI:** 10.1155/2020/8821181

**Published:** 2020-12-28

**Authors:** Anurak Wongta, Surat Hongsibsong, Somporn Chantara, Mookda Pattarawarapan, Ratana Sapbamrer, Korawan Sringarm, Zhen-Lin Xu, Hong Wang

**Affiliations:** ^1^Environmental Science Ph.D. Program, Faculty of Science, Chiang Mai University, Chiang Mai 50200, Thailand; ^2^School of Health Science Research, Research Institute for Health Science, Chiang Mai University, Chiang Mai 50200, Thailand; ^3^Cluster of Research and Development of Pharmaceutical and Natural Products Innovation for Human or Animal, Chiang Mai University, Chiang Mai 50200, Thailand; ^4^Environmental Chemistry Research Laboratory, Department of Chemistry, Faculty of Science, Chiang Mai University, Chiang Mai 50200, Thailand; ^5^Department of Chemistry and Center of Excellence for Innovation in Chemistry, Faculty of Science, Chiang Mai University, Chiang Mai 50200, Thailand; ^6^Department of Community Medicine, Faculty of Medicine, Chiang Mai University, Chiang Mai 50200, Thailand; ^7^Department of Animal and Aquatic Science, Faculty of Agriculture, Chiang Mai University, Chiang Mai 50200, Thailand; ^8^Guangdong Provincial Key Laboratory of Food Quality and Safety, College of Food Science, South China Agricultural University, Guangzhou 510642, China

## Abstract

Amyloid beta peptides (A*β*1-42) have been found to be associated with the cause of Alzheimer's disease (AD) and dementia. Currently, methods for detecting A*β*1-42 are complicated and expensive. The present study is aimed at developing an indirect competitive enzyme-linked immunosorbent assay (ic-ELISA) to detect A*β*1-42 by using a polyclonal antibody from alpaca, an application used in urine samples. The serum was collected from the alpaca after immunizing it with A*β*1-42 at 500 *μ*g/injection 5 times. The ic-ELISA was developed and showed a half-maximal inhibitory concentration (IC_50_) of 103.20 ng/ml. The limit of detection (LOD) was 0.39 ng/100 *μ*l. The cross-reactivity was tested with A*β*1-40 and 8 synthesized peptides that had sequence similarities to parts of A*β*1-42. The cross-reactivity of A*β*1-40 and peptide 1 (DAEFRHDSGYE) was 55% and 69.4%, respectively. The ic-ELISA was applied to analyze A*β*1-42 in the urine and precipitated protein urine samples. This method can be used for detecting a normal level of total soluble A*β* (approximately 1 ng in 5 mg of precipitated urine protein) and can be used for detecting the early stages of AD. It is considered to be an easy and inexpensive method for monitoring and diagnosing AD.

## 1. Introduction

Alzheimer's disease (AD) is an age-related chronic degenerative disease that damages the nervous system. It is mostly found in the elderly as a progressive neurodegenerative disease that predominantly affects cognitive functions [1]. The increasing number of AD patients is becoming an issue of serious concern due to the concomitant rise in medical and social costs. AD can be caused by several reasons, such as genetic causes, aging, malnutrition, immune system dysfunction, infectious agents, severe brain damage, congenital diseases (i.e., hypertension and diabetes), and the environment (i.e., exposure to pesticides or aluminum) [2, 3].

Amyloid beta peptides (A*β*) are neurotrophic and neurotoxic proteins that are used as a biomarker for detecting AD. Extracellular A*β* can lead to synapse loss and oxidative stress. Intracellular A*β* can increase the production of free radical species (ROS), causing increased levels of neurotoxicity and the death of neurons [4, 5]. A*β* is formed after sequential cleavage of the amyloid precursor protein (APP) by *β*-secretase, which removes the soluble APP*β* (sAPP*β*) and leaves a 99 amino acid C-terminal APP fragment (C99) in the cell membrane. Then, C99 is serial cleaved by *γ*-secretase at the *ε*-, *ζ*-, and *γ*-sites in the transmembrane domain (TMD), which releases the APP intracellular domain (AICD) into the cytosol and the 37-43 amino acid A*β* species into the extracellular space [6]. A*β* molecules can aggregate to form flexible soluble oligomers that may exist in several forms [7]. A*β* is the major constituent of the fibrils deposited into senile plaques and cerebral blood vessels of patients with AD. For A*β* levels under normal conditions, the daily excretion of intact soluble amyloid beta in the urine represents less than 1% of the circulating pool [8].

Several genetic, cell biology, biochemical, and animal studies support the concept that A*β* plays a central role in the development of AD pathology [9, 10]. The most common isoforms are A*β*1-40 and A*β*1-42 [11]. A*β*1-42 and A*β*1-43 are the major constituents of senile plaques and neurofibrillary tangles that occur in the hippocampus, neocortex, and amygdala of patients with AD [12]. A*β*1-42 is a good marker for the early stages of AD and is often detectable in several biological samples such as CSF, saliva, and urine in normal stages before the patient shows symptoms of AD, suggesting it is involved in the cause of AD [13–15].

Brain imaging techniques, which are crucial in the assessment of patients with AD, include fluoro-D-glucose integrated with positron emission tomography (FDG-PET) and amyloid positron emission tomography (amyloid PET) imaging [16, 17]. However, there are still many limitations of these methods, including cost, potential complications, the need for a specialist, and the requirement for high-performance equipment. While these conventional methods for identifying AD by detecting A*β*1-42 are still required and useful, they cannot detect the early stages of AD.

The immunoassay is a simple, economical technique with high sensitivity and specificity [18, 19] that can be developed and applied to biological samples. In the present day, immunoassays for human A*β* have been developed and can be purchased from pharmaceutical companies throughout the world. The most commercial enzyme-linked immunosorbent assay (ELISA) test kits are developed based on sandwich-ELISA, which requires two antibodies to detect the target antigen. They are used for serum, plasma, tissue homogenates, and cerebrospinal fluid (CSF). The sensitivity of commercial kits is a LOD between 4.73 and 1,000 pg/ml and a detection range between 7.8 and 480,000 pg/ml for all A*β*. A*β*1-42 can be present at a lower concentration, where the LOD is between 4.73 and 9.38 pg/ml. Conventional antibodies such as polyclonal antibodies (pAb) and monoclonal antibodies (mAb) from rodents are used in the test kits. The price of one test kit is in the range of 395-875 dollars for 96 tests [20], which is far too expensive to be widely used.

This study was conducted to develop ELISA for detecting A*β*1-42 in urine samples in which A*β* is found as a normal component, and the monomeric A*β* level is related to the clinical dementia rating score (CDR) [21].

## 2. Materials and Methods

### 2.1. Preparation of the Antibody

A single alpaca was raised at Nanchang (Jiangxi Province, People's Republic of China) and immunized subcutaneously 5 times at 2-week intervals with a 500 *μ*g/injection of A*β*1-42 to produce antibodies against A*β*1-42. The immunogen was prepared with A*β*1-42, which was purchased from Thermo Fisher (USA), dissolved in phosphate-buffered saline (PBS) pH 7.2, and gently mixed with an equal volume of Freund's Complete Adjuvant (Sigma-Aldrich, USA) to make an emulsion for the first immunization and Freund's Incomplete Adjuvant (Sigma-Aldrich, USA) to make an emulsion for the 2^nd^, 3^rd^, 4^th^, and 5^th^ immunization. The blood was collected from the jugular vein of the alpaca before the 4^th^ and 5^th^ injection and 1 week after the last immunization. The 5^th^ serum was used to develop the method in this study. The protocol of the experiment was approved by the animal ethical committee of South China Agricultural University, Guangdong, Guangzhou, China, and the study has been conducted in accordance with the guidelines for the animal screening of antiserum.

### 2.2. Indirect ELISA for the Titration of the Alpaca Antiserum

An indirect ELISA was used to determine the optimal dilution factor of the antibodies that were later used in ic-ELISA procedure [22, 23]. Standard A*β*1-42 was serially diluted into coating buffer (carbonate buffer, pH 9.6), producing 11 concentrations of A*β*1-42, and coated on Maxisorp microwell plates (Thermo Fisher Scientific, Denmark) at 4°C for 16 hours overnight. After incubation, the plate was washed 4 times with 400 *μ*l/well PBST (PBS with 0.05% Tween) by a microplate washer (Thermo Fisher Scientific, Finland) and dried by patting with paper towels. After washing, the plate was blocked with 200 *μ*l/well of 1% skim milk in PBS as a blocking solution and incubated for 1 hour at 25°C. Then, the blocking solution was discarded, and the plate was dried by patting with paper towels. Alpaca antiserum, which was prepared with 8 dilutions from 1 : 1,000 to 1 : 32,000, was added to the wells and incubated for 1 hour at 25°C. Then, the plate was washed as in the previous washing procedure. In the next step, 100 *μ*l of goat anti-llama IgG labeled with horseradish peroxidase (1 : 5,000 in PBST) (Abcam, USA) was added to each well and incubated for 1 hour at 25°C. Then, the washing procedure was repeated. For determination, 100 *μ*l of TMB substrate (12.5 ml citrate buffer pH 4, 200 *μ*l stock TMB, and 50 *μ*l of 1% H_2_O_2_) was added and rested in the dark at 25°C for 10 min. The reaction was stopped with 2 M H_2_SO_4_. The optical density (OD) was measured at 450 nm in a multiscan spectrophotometer (Tecan, Austria) to evaluate the optimal dilution of the alpaca serum and the concentration of coating [24].

### 2.3. Indirect Competitive ELISA for Quantification of A*β*1-42

The ic-ELISA assay was performed with alpaca antiserum (1 : 1,000 in PBST), and the competition between the free standard A*β*1-42 and the coated antigen (A*β*1-42) was observed in order to determine the concentration of the free standard. The microwell plate was coated at 4°C for 16 hours overnight with 100 *μ*l/well of 3 concentrations of coating antigen (A*β*-42, 2-4 *μ*g/ml in coating buffer). The plate was then washed and blocked as described above. An equal volume of standard A*β*-42 was added to the alpaca antiserum in a u-shaped plate at 25°C for 1 hour. After the blocking solution was discarded and dried, the mixture of standard A*β*1-42 and alpaca antiserum was added in triplicate to the wells of each coating antigen. After the plate was incubated for 1 hour at 25°C and washed, anti-llama IgG labeled with horseradish peroxidase (1 : 5,000 in PBST) and TMB substrate were added following the ELISA protocol, as described above.

The specificity and sensitivity of this method were measured with alpaca antiserum (1 : 1,000 in PBST), and the competition between the free standard A*β*1-42 with the coated antigen (A*β*1-42) was observed. The microwell plate was coated at 4°C for 16 hours overnight with 100 *μ*l/well of 2 *μ*g/ml A*β*1-42 in coating buffer. Twelve concentrations of competitive A*β*1-42 were prepared (0-4,000 ng/ml, twofold dilution), and ic-ELISA was performed with 1 : 1,000 (v/v) dilution of alpaca antiserum, as described above.

The calibration curve was obtained and calculated as IC_50_. The limit of detection (LOD) was calculated by using the formula [25] as follows:

(1) $$\begin{array}{c} \text{LOD}=B_{0}–3\text{SD}, \end{array}$$

where *B*_0_ is the optical density (OD) of blank (no A*β*1‐42) and SD is the standard deviation of *B*_0_.

### 2.4. Assessment of the Cross-Reactivity of A*β*1-42 ic-ELISA

Standard A*β*1-40, A*β*1-42, and 8 synthesized A*β* peptides (A*β*1-8, A*β*7-15, A*β*14-21, A*β*20-27, A*β*26-33, A*β*32-39, A*β*35-42, and A*β*36-43) were tested in the ic-ELISA assay as described above. Calibration curves were obtained. The cross-reactivity (CR) values were calculated by using the formula [26] as follows:

(2) $$\begin{array}{c} \text{Cross}‐\text{reactivity rate }\left(\%\right)=\frac{{\text{IC}}_{50}\text{ of A}\beta 1-42}{\left({\text{IC}}_{50}\text{ of the structural}‐\text{related protein}\right)\times 100}, \end{array}$$

where IC_50_ is the half-maximal inhibitory concentration.

### 2.5. Preparation of the Urine Protein

A pool of urine was prepared from 10 healthy donors (5 male and 5 female) and used in this study. Due to the small amount of A*β*1-42 in urine, the concentration of the urine proteins was increased by a precipitation technique. Urine protein was prepared by the salting out technique, which is the most common method used to precipitate proteins. Ammonium sulfate ((NH_4_)_2_SO_4_) was used to compress the solvation layer and increase the protein–protein interactions. The increasing salt concentration caused the protein to separate and fall to the bottom of the sample solution by aggregation and precipitation activities [27]. The precipitation was performed with 31.77 g of ammonium sulfate added to 100 ml urine (to reach 50% saturation) and well-mixed by a magnetic stirrer for 1 hour on ice. Then, the mixture was centrifuged 4,000 rpm for 1 hour at 4°C. The supernatant was discarded; then, the protein pellet was resuspended in 1 ml PBS buffer (pH 7.2) and kept at 4°C.

### 2.6. Assessment of the Matrix Effect on A*β*1-42 ic-ELISA Calibration Graph

The effects of urine and precipitated urine protein on the performance of the ic-ELISA were assessed. Twelve dilutions of each urine sample (1 : 1-1 : 5,120 in PBS) and precipitated urine protein (0.001-4.56 mg/ml) were determined by ic-ELISA, as described above. The nonmatrix effect was determined by the same value OD of the sample dilution and the OD of the PBS sample.

Calibration curves of the A*β*1-42 in urine and precipitated urine protein were obtained. Twelve concentrations of A*β*1-42 (0–8,000 ng/ml) were spiked into 1 : 160 urine in PBS and 0.456 mg/ml precipitated urine protein, and then, ic-ELISA was performed as described above. LODs were calculated as described above.

The correlation curves were obtained by comparing the concentration of spiked A*β*1-42 samples and the detected concentration in the calibration curves.

## 3. Results

### 3.1. Development of ic-ELISA

**Figure 1:**
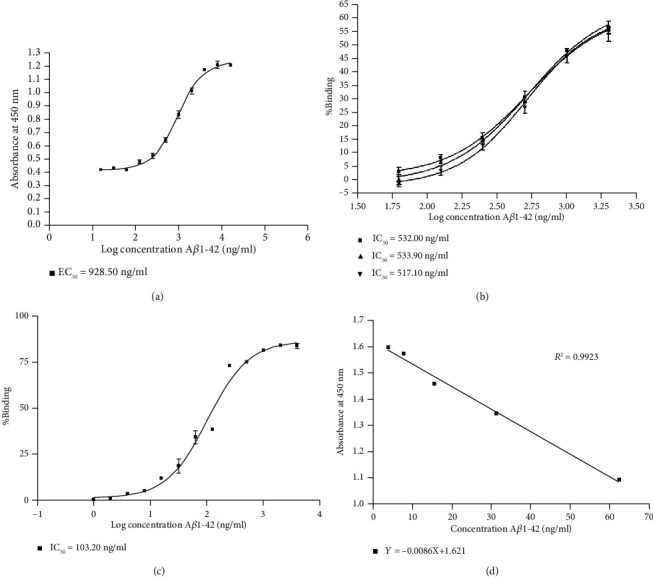
Development of A*β*1-42 detection. (a) Indirect ELISA calibration curves for A*β*1-42 using 1 : 1,000 (v/v) dilution of alpaca antiserum. (b) Optimization of coating antigen using indirect competitive ELISA. The percent binding showed a competition percentage of 6 concentrations of competitive A*β*1-42 (62, 125, 250, 500, 1,000, and 2,000 ng/ml) to a 1 : 1,000 (v/v) dilution of alpaca antiserum at 3 concentrations of coating antigen (■ = 2,000, ▲ = 3,000, and ▼ = 4,000 ng/ml of A*β*1-42 in coating buffer). (c) ic-competitive ELISA calibration curves for A*β*1-42 using a 1 : 1000 (v/v) dilution of alpaca antiserum. The calibration curve was obtained using analyte standards prepared in PBS buffer. (d) ic-competitive ELISA calibration curves obtained by OD.

To develop the ic-ELISA for the detection of A*β*1-42, alpaca antiserum was tested using indirect ELISA in the first step to determine the optimal concentration of coating antigen and the optimal dilution of antiserum. Six concentrations of A*β*1-42 in coating buffer were coated on the wells, and six serial dilution ratios from 1 : 1,000 to 1 : 32,000 of alpaca antiserum were investigated. The 1 : 1,000 dilution showed the highest binding sensitivity among all coating antigen concentrations (Table 1). Then, sensitivity of the indirect ELISA was tested with 15 concentrations of A*β*1-42. The half-maximal effective concentration (EC_50_) of a 1 : 1,000 (v/v) dilution of alpaca antiserum was 928.40 ng/ml as shown in Figure 1(a).

As the second step, the ic-ELISA was used to optimize the concentration of coating antigen (A*β*1-42). Three concentrations of A*β*1-42, prepared at 2,000, 3,000, and 4,000 ng/ml in the coating buffer, were used as the coating antigen, and ic-ELISA was performed with a 1 : 1,000 (v/v) dilution of alpaca antiserum, as described above. The coated concentration of 2,000 ng/ml A*β*1-42 was selected since it showed the same half-maximal inhibitory concentration (IC_50_) as the above concentrations (Figure 1(b)).

For the third step, the sensitivity of this method was determined. The IC_50_ of ic-ELISA was 103.20 ng/ml as shown in Figure 1(c). The LOD of this method was 0.39 ng/100 *μ*l, calculated by the formula and OD calibration curve shown in Figure 1(d).

### 3.2. Cross-Reactivity of the Assay

The specificity of the developed ic-ELISA was assessed by measuring the cross-reactivity toward the same structural proteins as A*β*1-42, using A*β*1-40, and 8 synthesized A*β* peptides (GenScript, China) in the test (the sequence of peptides is shown in Table 2). The data within a range of 12 concentrations of the A*β*1-42 standard (0–4,000 ng/ml, twofold dilution in PBST) were used for the calculation of cross-reactivity. A*β*7-15, A*β*14-21, A*β*20-27, A*β*26-33, A*β*32-39, A*β*35-42, and A*β*36-43 showed no detectable cross-reactivity. However, A*β*1-8 showed 69.40% and A*β*1-40 showed 55.00% cross-reactivity as shown in Table 2, suggesting both can have some contribution to the measurement of the A*β*1-42 level with this method.

### 3.3. Matrix Effect on the A*β*1-42 ic-ELISA

**Figure 2:**
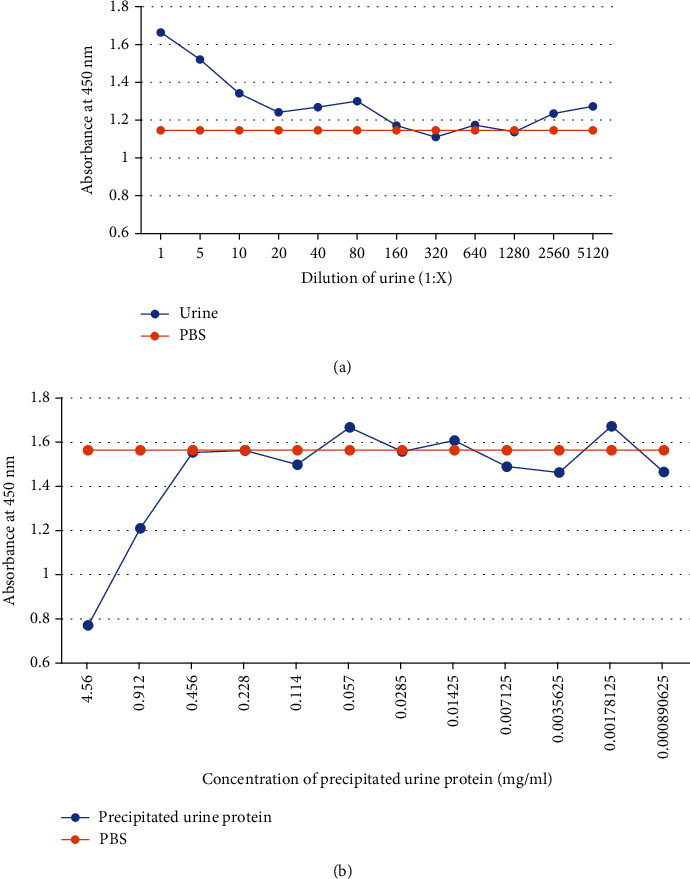
Observation of the urine matrix effect using ic-ELISA. (a) The curve was obtained using analyte 12 dilutions of pooled urine prepared in PBS buffer (1 : 1–1 : 5120). (b) The curve was obtained using analyte 12 concentrations of precipitated urine protein prepared in PBS buffer (0.001-4.56 mg/ml).

**Figure 3:**
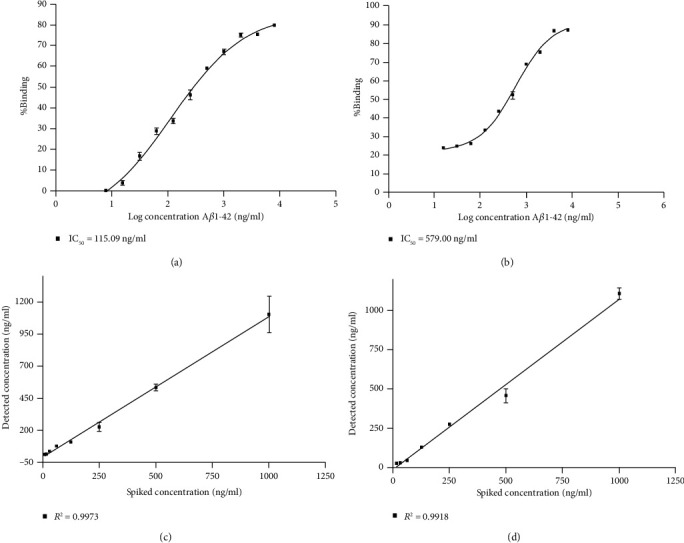
The ic-ELISA calibration curves for A*β*1-42 using a 1 : 1000 (v/v) dilution of alpaca antiserum were obtained using analyte standards prepared in 0.456 mg/ml precipitated urine. (c) The correlation curve of the detected and spiked concentrations of A*β*1-42 in 1 : 160 diluted urine. (d) The correlation curve of the detected and spiked concentrations of A*β*1-42 in 0.456 mg/ml precipitated urine.

The developed ic-ELISA was performed by using a 1 : 1,000 (v/v) dilution of alpaca antiserum in 12 diluted samples (1 : 1–1 : 5120 in PBS) of pooled urine and 12 diluted samples of precipitated urine protein (urine protein = 0.001‐4.56 mg/ml) that were used as the matrix antigen of competition with coated A*β*1-42. The results had no matrix effect OD at 1 : 160 for the pooled urine, as shown in Figure 2(a), and 0.456 mg/ml for the urine protein, as shown in Figure 2(b). The calibration curve was obtained using analyte standard A*β*1-42 prepared in 1 : 160 diluted pool urine and 0.456 mg/ml precipitated urine protein. The results showed an IC_50_ = 115.90 ng/ml for 1 : 160 pooled urine, as shown in Figure 3(a), and 579.00 ng/ml for precipitated urine protein, as shown in Figure 3(b). The LOD of this method was 16.83 ng/ml for the pooled urine sample and 139.60 ng/ml for the precipitated urine protein. The correlation of the detected and spiked concentrations of A*β*1-42 in the samples by using the ic-ELISA calibration curve showed *R*^2^ = 0.9973 for pooled urine (in the range 7.80-1000.00 ng/ml A*β*1-42) as shown in Figure 3(c) and *R*^2^ = 0.9918 for the precipitated urine protein (in the range 15.63-1000.00 ng/ml A*β*1-42) as shown in Figure 3(d).

## 4. Discussion

We propose a new alternative method for the detection of A*β* in human biological samples, an ic-ELISA for the detection of A*β*1-42 in urine samples that we have developed. Most commercial ELISA test kits for A*β* in humans are developed based on sandwich-ELISA, which requires two antibodies for detection, and only a few commercial test kits can detect A*β* in urine. One advantage of our method is that it only requires one specific antibody and can be detected in urine samples. Another advantage of using a single antibody is reducing nonspecific bounding since other substances may affect each antibody. The commercial kits have a LOD between 4.73 and 1,000 pg/ml, and their detection range is between 7.8 and 480,000 pg/ml for all A*β*. A*β*1-42 shows a lower concentration, where LOD is usually between 4.73 and 9.38 pg/ml. Our method shows that the concentration of the LOD for A*β*1-42 is higher than the commercial kit at 3.90 ng/ml (390 pg/well), based on the levels of A*β*1-42 in human biological samples that are regularly monitored at relatively low levels: 750 pg/ml for CSF samples [28] and 17.65 pg/ml for serum samples [29]. So, we are not able to use the commercial methods to detect A*β*1-42 in these samples.

However, our method showed 69.4% and 55% cross-reaction to the synthesized A*β*1-8 and A*β*1-40, respectively, as expected, since A*β*1-40 has a sequence close to A*β*1-42. Synthesized A*β*1-8 also has a sequence present at the beginning of every A*β*. The LOD of this method cannot detect A*β*1-42 in urine samples. However, due to the high cross-reactivity, the method can be developed to detect total A*β* in urine and could be used to detect total A*β* in other biological samples.

In urine samples, the LOD was 16.83 ng/ml and 139.60 ng/ml for precipitated urine protein. Since urine contains inorganic ions (K +, Na +, Cl −, and Ca2 +), organic molecules such as creatinine, urea, and uric acid, and more than 1,500 proteins, mainly extracellular, membranes, cell membranes, and cell debris, the higher LOD may come from the matrix in urine, which can interrupt the binding of antigens with antibodies used in the ELISA assays [30]. Another limitation of commercial ELISA kits is the price; most are expensive and cannot be routinely used for early detection in people. The yields of pAb from rodents are limited due to the size of the animal. In this study, the antibody was produced in an alpaca, whose blood can be collected in larger quantities, which allows our method to be developed at a lower cost.

A previous study reported that A*β* is removed from circulation by the capillary beds of the kidneys, liver, and gastrointestinal tract, as well as through the skin and excreted in the urine [31]. According to studies from Takata et al., A*β* has a correlation with the clinical dementia rate (CDR) of people with AD and mild cognitive impairment (MCI), with an increasing value of A*β* in mild AD (CDR 0.5 to 1) and a decreasing value in more severe AD (CDR 2 to 3), since aggregated A*β* plaques might not be able to be eliminated via normal clearance routes in severe cases. The detection sensitivity of their method was 40 pg/ml, which still cannot be quantified [21]. Since our new ic-ELISA method can detect this amount of A*β* in urine samples, it can be used as a preliminary assessment for AD.

A*β* peptides are a normal component of human urine as identified in the study of Ghiso et al. [8], who conducted immunoprecipitation experiments with anti-A*β* antibodies followed by detection and identification by immunoblots and MALDI mass spectrometry. For A*β* levels in normal donors (proteinuria < 5 mg/dl), the excretion of soluble A*β* was calculated at 0.81 ± 0.26 ng/5 mg of urinary proteins (13 ± 4 ng/24 h) [8]. Therefore, at this A*β* level, our method could measure the level of monomer A*β* in the urine. By applying urine sample preparation methods, such as precipitation techniques to increase the concentration of A*β* in the urine sample, we can use 1 dl of urine to prepare 5 mg urine protein, which can reach approximately 1 ng of A*β* in normal donors. In addition, from the urine matrix tests, our method found an effect of the pooled urine at under 1 : 160 dilution (urine protein < 32.25 ng in 100 *μ*l sample). This means that it is not possible to use this method to determine A*β* in urine samples without a sample pretreatment process. However, since precipitated urine protein has shown nonmatrix effect results at 0.456 mg/ml (urine protein = 0.05 mg in 100 *μ*l sample) and unidentified inhibition results of 4.56 and 0.91 mg/ml (urine protein = 0.46 and 0.09 mg in 100 *μ*l sample), it is possible to develop this method to detect amyloid in urine samples in combination with appropriate urine protein sample preparation.

The serum and CSF sample collection requires invasive methods and can be harmful to participants, while urine samples can be easily and not harmfully collected. The results can be corrected referencing the concentration of A*β* against urine creatinine and reported as microgram per milliliter urine or microgram per gram creatinine, as reported in several previous studies.

Most of the immunoassay methods were developed based on conventional antibodies and have some limitations, i.e., the lack of specificity of pAb and complicated production of mAb [32]. However, the production of a single-domain antibody (sdAb) from a phage display technique could provide more sensitivity, specificity, and stability of the antibody. In addition, a large number of antibodies can be produced by these methods [33]. An sdAb is an antibody fragment consisting of a single monomeric variable antibody domain, unlike a whole antibody, which can selectively bind to a very specific antigen. The sdAb could be produced from both conventional antibodies [34] and heavy chain antibodies from camelids [35].

In our study, an alpaca was immunized with A*β*1-42 to produce antibodies. For our novel ic-ELISA for A*β*, pAb was applied due to its convenience in the development of this method of examination, but sdAb for A*β* can be developed in the future.

## 5. Conclusions

The developed ic-ELISA indicated sensitivity for A*β*1-42 and peptides having a similar structure. A*β*1-40 showed 55% cross-reactivity, and the synthesized A*β*1-8, which consists of the same beginning sequence for all A*β*, showed 69.4% cross-reactivity. In addition, the method showed no cross-reactivity to other synthesized A*β* peptides, which have the same sequence as A*β*1-42 from different segments of the sequence. We can presume that the binding site of the antibody has the same sequence as A*β*1-8 (DAEFRHD). The assay, as described in this study, has great potential to detect A*β*1-42 protein in cases of high-risk and early stages of AD, with an easy and rapid method when applied to a urine sample. Moreover, this assay can be widely used at a low cost and applied to other types of biological samples.

## Figures and Tables

**Table 1:** Results of the ELISA checker-board titration test of the binding of 6 concentrations of A*β*1-42 and 6 serially diluted antiserums by the indirect ELISA method.

Antiserum (1 : X)	OD at 450 nm					
	Concentration of coated A*β*1-42 (ng/ml)					
	4000	2000	1000	500	250	125
1,000	1.053	0.938	0.852	0.74	0.644	0.525
2,000	0.929	0.799	0.686	0.56	0.443	0.368
4,000	0.821	0.622	0.475	0.379	0.272	0.217
8,000	0.659	0.441	0.342	0.235	0.178	0.146
16,000	0.456	0.278	0.203	0.149	0.113	0.099
32,000	0.293	0.171	0.13	0.098	0.081	0.073

**Table 2:** Cross-reactivity of synthesized peptides and two beta amyloid proteins.

Sample	Sequence	IC_50_ (ng/ml)	% cross-reaction
A*β*1-42	DAEFRHDSGYEVHHQKLVFFAEDVGSNKGAIIGLMVGGVVIA	103.20	100.00
A*β*1-8	DAEFRHDS	148.80	69.40
A*β*1-40	DAEFRHDSGYEVHHQKLVFFAEDVGSNKGAIIGLMVGGVV	187.70	55.00
A*β*7-15	DSGYEVHHQ	>4000	-
A*β*14-21	HQKLVFFA	>4000	-
A*β*20-27	FAEDVGSN	>4000	-
A*β*26-33	SNKGAIIG	>4000	-
A*β*32-39	IGLMVGGV	>4000	-
A*β*35-42	MVGGVVIA	>4000	-
A*β*36-43	VGGVVIAT	>4000	-

## Data Availability

All data were shown in the manuscript.
